# Trends of Acupuncture Therapy on Depression From 2011 to 2020: A Bibliometric Analysis

**DOI:** 10.3389/fpsyg.2021.721872

**Published:** 2021-10-13

**Authors:** Hongchun Xiang, Jing Li, Bocun Li, Qian Tan, Guowei Cai

**Affiliations:** Department of Acupuncture and Moxibustion, Union Hospital, Tongji Medical College, Huazhong University of Science and Technology, Wuhan, China

**Keywords:** acupuncture, depression, bibliometric analysis, CiteSpace, Web of Science

## Abstract

**Objectives:** The purpose of this study was to explore the current status and trends of acupuncture for depression in the last decade and provide new insights for researchers in future studies.

**Methods:** The articles regarding acupuncture treatment for depression published between 2011 and 2020 were extracted from the Web of Science Core Collection. We used CiteSpace to analyze data on publications, countries, institutions, cited journals, cited authors, cited references, keywords, and citation bursts about acupuncture and depression.

**Results:** A total of 1,032 publications were obtained from 2011 to 2020. We identified the most prolific journals, countries, institutions, and authors in the field of acupuncture for depression in the last decade. The most prolific country and institutions were the People's Republic of China and KyungHee University, respectively. Evidence-based Complementary and Alternative Medicine was the most prolific and cited journal. The author with the highest centrality was Zhangjin Zhang, and the author with the most publications was Park Hi-Joon. The keyword “cognitive behavioral therapy” was first for research developments with the highest citation burst. The five hot topics in acupuncture on depression were “acupuncture,” “depression,” “electro-acupuncture,” “quality of life,” and “anxiety.”

**Conclusions:** The results from this bibliometric study provide insight into the research trends in acupuncture therapy for depression, and the current status and trends of the past decade, which may help researchers determine the current status, hotspots, and frontier trends in this field.

## Introduction

Depression is a major disease burden in the world. The worldwide prevalence of the major depressive disorder is estimated at 4.7%, with an annual incidence rate of about 3% (Ferrari et al., 2013). Depression is an important factor in the quality of life and survival, accounting for about 50% of all psychiatric outpatients and 12% of all inpatients (Kuo et al., 2015). In total, 5.8% of men and 9.5% of women experience depressive episodes in any year of their lifetime (Li et al., 2016). The WHO lists depression as the fourth leading cause of disability worldwide (Guo et al., 2019). China is also facing the challenge of a rising incidence of depression. In China, depression has become one of the main causes of disability (Yang et al., 2013; Jin et al., 2019). Depression has a major impact on the quality of life of patients and imposes a significant economic burden on them (Hofmann et al., 2017).

There are three main treatments for depression: antidepressants and other auxiliary drugs, evidence-based psychotherapy (such as cognitive-behavioral therapy and interpersonal psychotherapy), and somatic non-drug therapy, including electroconvulsive therapy, repetitive transcranial magnetic stimulation, and vagus nerve stimulation (Gartlehner et al., 2017). Patients with depression may be more likely to choose non-drug treatments because antidepressant drugs pose a significant risk of adverse events. Up to 63% of the patients who take second-generation antidepressants experience adverse events; 7–15% stop taking the drug treatment due to these events (Gartlehner et al., 2008). Addiction to antidepressants is also a common concern (Tirado Munoz et al., 2018; Zhou et al., 2020). In one report, 50% of self-reported depressive disorder patients opted for complementary or alternative medical treatments (Ashraf et al., 2021). Acupuncture is not only traditional Chinese medicine but also a part of the current medical system of China. Some meta-analyses showed that acupuncture is a safe and effective method for the treatment of depression (Li et al., 2018; Armour et al., 2019), with relatively few adverse effects (Smith et al., 2018). More research effort is being devoted to the application of acupuncture for the treatment of depression (Li R. et al., 2020). However, little attention has been paid to topic hotspots and trends in acupuncture for depression.

The Web of Science database contains more and more works of literature every year. Meanwhile, it remains a question whether the number of publications in acupuncture treatment for depression changed over time. Which countries, institutions, and authors are most active in this study? What are the hot topics and research trends in this research field? A bibliometric analysis is a statistical analysis and quantitative tool to study publications. The cross-science of quantitative analysis can show the global research trends and topic hotspots of a research field (Leefmann et al., 2016; Ozsoy and Demir, 2018). Pei et al. (2019) performed a global bibliometric analysis based on the Web of Science database to estimate the trends of acupuncture therapy for insomnia in the last 20 years (1998–2018). Li W. et al. (2020) showed a global bibliometric analysis based on the Web of Science assessing trends in acupuncture therapy for knee osteoarthritis in the last decade (2010–2019). The previous retrospective analyses of acupuncture treatment for depression were mainly literature reviews and meta-analyses. However, a specific bibliometric analysis of acupuncture for depression has not yet been performed.

In this study, CiteSpace was searched to obtain data on the hot topics and research trends in the use of acupuncture treatment for depression during the last decade for the first time.

## Methods

### Data Acquisition

All publications were obtained from the Web of Science Core Collection (https://www.webofscience.com/wos/woscc/advanced-search) (Clarivate 30 Thomson Place, 36T3 Boston, MA 02210) in this study on May 5, 2021. The search terms used were “depression” and “acupuncture therapy,” and all extracted studies were published in the last decade. Here are the search strategies: 1# TS=((Acupunctur^*^) OR (Acupunctur^*^ Treatment^*^) OR (Acupunctur^*^ Therap^*^) OR (body Acupunctur^*^) OR (Needle^*^ Acupunctur^*^) OR (Manual^*^ Acupunctur^*^) OR (Acupunctur^*^ Point^*^) OR (Electroacupunctur^*^) OR (Warm^*^ Acupunctur^*^) OR (electr^*^-acupunctur^*^)), 2# TS=(depression OR depressions OR depressed OR despondent OR gloomy OR depressive OR antidepressant OR antidepressants); Indexes = Web of Science Core collection, namely: Science Citation Index-Expanded (SCIE); Social Sciences Citation Index (SSCI); Arts and Humanities Citation Index (A&HCI); Emerging Sources Citation Index (ESCI); Proceedings Citation Index Science (CPCI-S); Conference Proceedings Citation Index Social Science & Humanities (CPCI-SSHI); Book Citation Index– Science (BKCI-S); Book Citation Index– Social Sciences & Humanities (BKCI-SSH); timespan = 2011–2020, 1# AND 2#.

### Analysis Method

We used the Citespace 5.1.R6 SE ((c)2003-2018 Chaomei Chen. All rights reserved.) for the bibliometric analysis. First, we performed a series of heuristics and manual checks to disambiguate the names of the top authors that appear in several orthogonal variants (for example, Yuan Zhang, Ying Zhang, and Yue Zhang were combined into one author name ZHANG Y). The results showed the trends in annual publication counts and the most prolific journals, authors, institutions, and countries. Co-occurrence analysis of keywords, authors, references, and institutions was also performed. The trends of acupuncture treatment for depression were subjected to a visualization analysis.

The CiteSpace parameters were as follows: time slicing, 2011–2020; years per slice, 1; source terms, all options; node selection type, one at a time; pruning, pathfinder. Graphs of nodes and linkages were generated. Each node represented an element, such as author, institution, and country, among others. Nodes with different colors between the inside and the outside parts represented the period 2011–2020. Lines between nodes indicated the co-occurrence of a co-citation. Purple circles indicated centrality, and nodes with high centrality were considered more important.

## Results

### Annual Numbers of Publications

**Figure 1:**
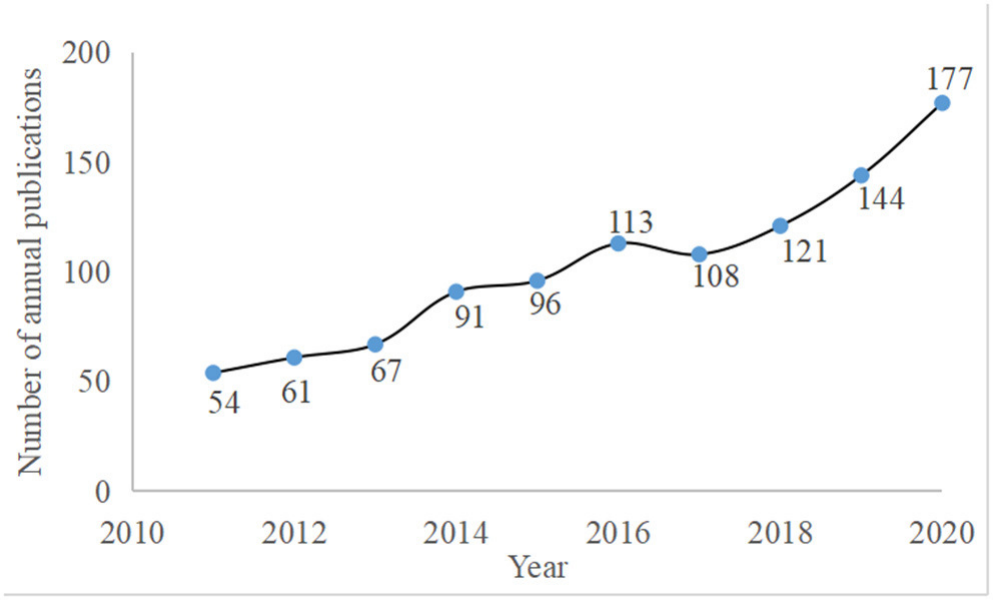
The annual number of publications on acupuncture for depression from 2011 to 2020.

A total of 1,032 articles were extracted by searching the Web of Science Core Collection. The document types of interest were Articles, Review Articles, Letters, Editorial Material, Meeting Abstracts, Proceedings Papers, News Items, Book Chapters, and Early Access. The number of specific articles published each year is shown in Figure 1. In the past 10 years, the annual number of publications on acupuncture treatment for depression fluctuated slightly. From 2011 to 2016 and from 2018 to 2020, the number of publications showed increasing trends. However, the number of publications decreased from 113 in 2016 to 108 in 2017. In 2018, the number of publications increased again to 121. The lowest number of publications was in 2011 (*n* = 54). The highest number of publications was in 2020 (*n* = 177). In recent years, acupuncture treatment has received increasing attention, and the efficacy of acupuncture for the treatment of depression has also been the subject of more research.

### Analysis of Journals and Cited Journals

**Figure 2:**
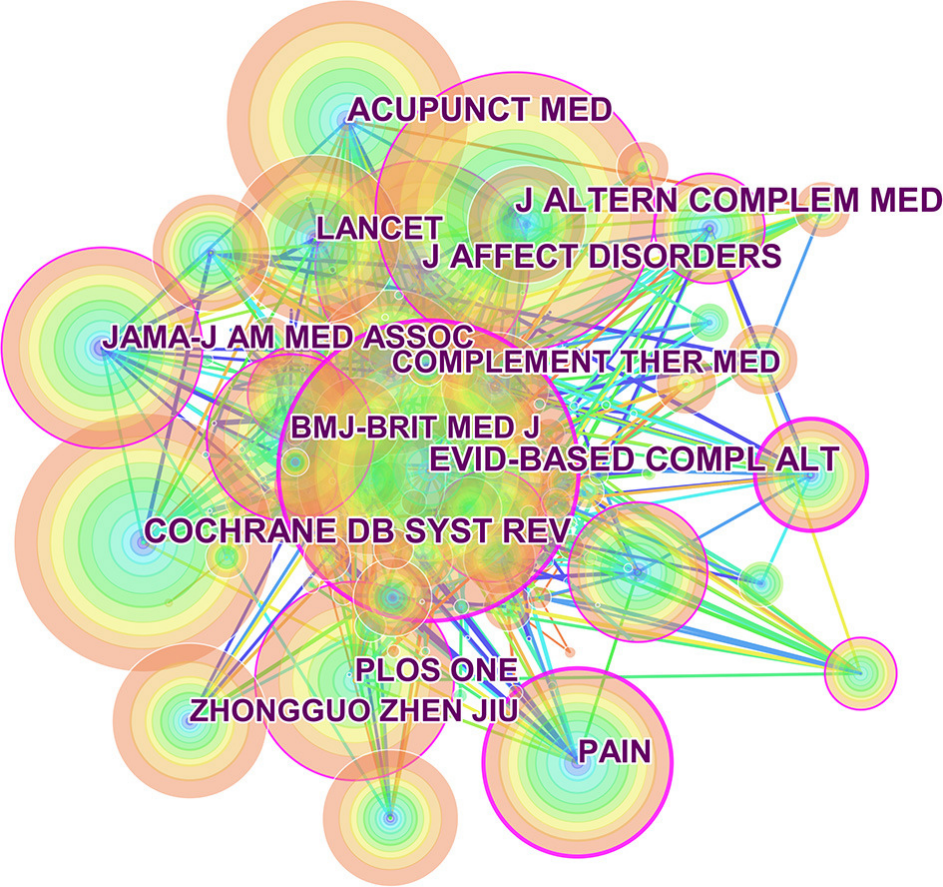
Cited journal maps related to acupuncture treatment for depression research from 2011 to 2020.

The top 10 journals on acupuncture for depression are listed in Table 1. *Evidence-Based Supplementary and Alternative Medicine* was the most prolific journal, with 68 articles, followed by *Trials* with 59 articles. A journal map was generated based on 9,662 references using CiteSpace (Figure 2). The top five most-cited journals and data on co-citation centrality are shown in Table 2. The top-ranked cited-journals for frequency and centrality were *Evidence-Based Supplementary and Alternative Medicine* (*n* = 425) and *Pain* (0.26), respectively. A systematic review and meta-analysis of 425 records showed that acupuncture can effectively alleviate depression. The meta-analysis included 13 randomized controlled trials involving 1,046 subjects. The scores on the 17-item Hamilton Rating Scale for Depression showed that acupuncture combined with selective serotonin reuptake inhibitors (SSRIs) was more effective than a single-drug treatment (Chan et al., 2015).

**Table 1:** Top 10 scholarly journals related to acupuncture on depression.

**Rank**	**Publications**	**Journal**	**IF (2020)**	**Rank**	**Publications**	**Journal**	**IF (2020)**
1	68	Evidence Based Complementary and Alternative Medicine	2.629	6	23	BMC Complementary and Alternative Medicine	3.659
2	59	Trials	2.279	7	23	Complementary Therapies in Medicine	2.446
3	43	Medicine	1.889	8	22	European Journal of Integrative Medicine	1.314
4	30	Acupuncture in Medicine	2.267	9	18	Explore-the Journal of Science and Healing	1.775
5	27	Journal of Alternative and Complementary Medicine	2.579	10	17	Medical Acupuncture	0

**Table 2:** Top five frequencies and centrality of cited journals related to acupuncture on depression.

**Rank**	**Cited journal**	**Frequency**	**Rank**	**Cited journal**	**Centrality**
1	Evidence-Based Complementary and Alternative Medicine	425	1	Pain	0.26
2	Journal of Alternative and Complementary Medicine	397	2	Evidence-Based Complementary and Alternative Medicine	0.21
3	Cochrane Database of Systematic Reviews	377	3	American Journal of Psychiatry	0.21
4	Acupuncture in Medicine	349	4	British Medical Journal	0.18
5	Journal of Affective Disorders	268	5	Neuroscience Letter	0.16

### Distribution of Countries and Institutions

**Figure 3:**
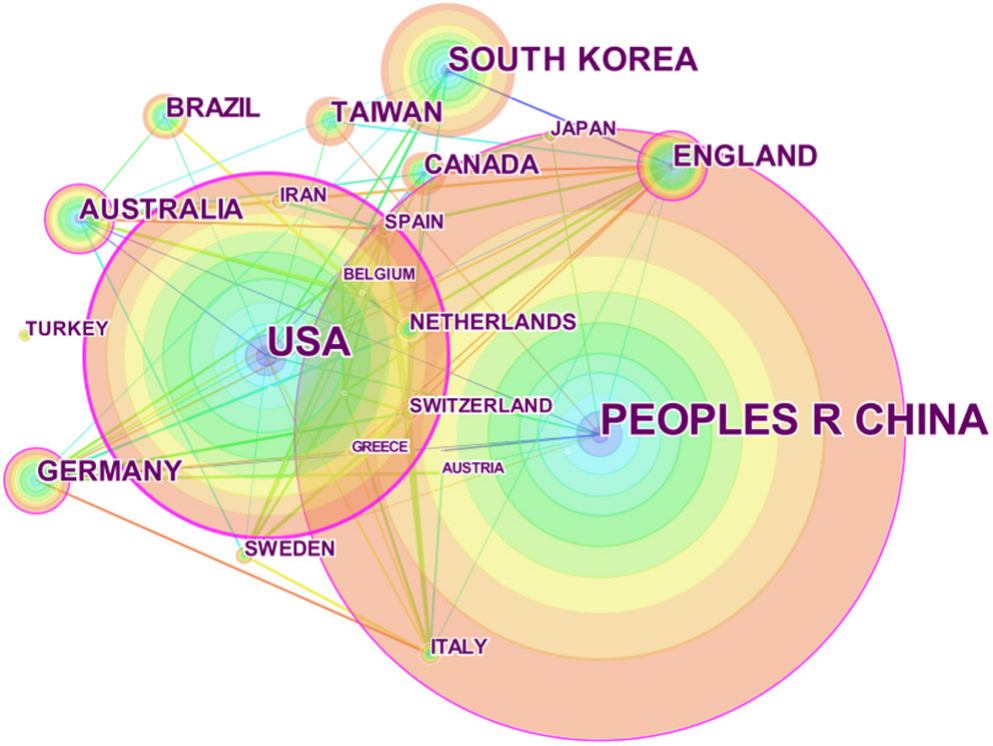
Map of countries researching acupuncture for depression from 2011 to 2020.

A country distribution map was generated with CiteSpace, and 32 nodes and 79 links were present in the network (Figure 3). In total, 1,032 publications were published by researchers in 32 countries. Most publications come from China, where acupuncture originated. In the United States, South Korea, England, and Australia, researchers pay more attention to acupuncture as a specific treatment for depression (Table 3).

**Table 3:** Top 10 publications and centrality of countries related to acupuncture on depression.

**Rank**	**Publications**	**Countries**	**Rank**	**Centrality**	**Countries**
1	406	China	1	0.26	United States
2	248	United States	2	0.18	China
3	95	South Korea	3	0.17	Australia
4	52	United Kingdom	4	0.16	United Kingdom
5	50	Australia	5	0.14	Germany
6	48	Germany	6	0.06	Spain
7	40	Taiwan	7	0.05	Canada
8	35	Brazil	8	0.05	Italy
9	34	Canada	9	0.04	Switzerland
10	18	Netherlands	10	0.03	Sweden

**Figure 4:**
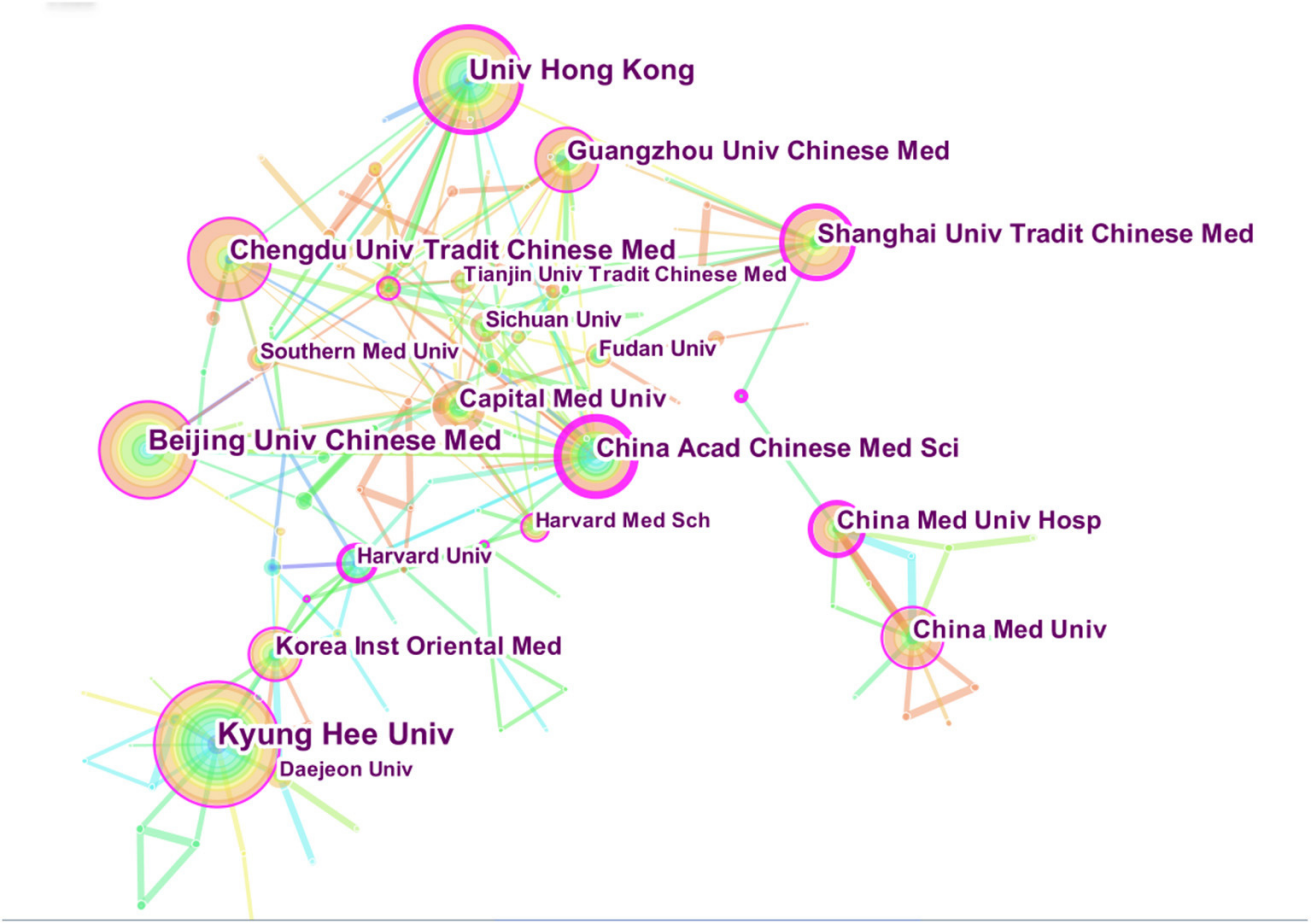
Map of institutions researching acupuncture for depression from 2011 to 2020.

The distribution map of the institutions consisted of 153 nodes and 222 links (Figure 4). A total of 153 institutions provided studies on acupuncture for depression. The top five most prolific institutions were KyungHee University, University of Hong Kong, Beijing University of Chinese Medicine, Chengdu University of Traditional Chinese Medicine, and China Academy of Chinese Medical Sciences. Meanwhile, the top 5 institutions in terms of centrality were China Academy of Chinese Medical Sciences, Shanghai University of Traditional Chinese Medicine, Harvard University, University of Hong Kong, and the University of Illinois. In particular, the China Academy of Chinese Medical Sciences was the institution with the highest centrality, suggesting that it is an important institution in the research on acupuncture for depression (Table 4). Based on the numbers of publications and centrality, the institutions from China, the United States, South Korea, and other countries pay the most attention to acupuncture on depression.

**Table 4:** Top 10 publications and centrality of institutions related to acupuncture on depression.

**Rank**	**Publications**	**Institutions**	**Rank**	**Centrality**	**Institutions**
1	55	Kyung Hee University	1	0.49	China Academy of Chinese Medical Sciences
2	46	University of Hong Kong	2	0.34	Shanghai University of Traditional Chinese Medicine
3	43	Beijing University of Chinese Medicine	3	0.24	Harvard University
4	37	Chengdu University of Traditional Chinese Medicine	4	0.23	University of Hong Kong
5	33	China Academy of Chinese Medical Sciences	5	0.22	University of Illinois
6	31	Shanghai University of Traditional Chinese Medicine	6	0.21	China Medical University, Hospital 1
7	28	Guangzhou University of Chinese Medicine	7	0.2	Kyung Hee University
8	27	China Medical University	8	0.18	Korea Institute for Oriental Medicine
9	26	Capital Medical University	9	0.18	University of Maryland
10	25	Korea Institute for Oriental Medicine	10	0.14	Chengdu University of Traditional Chinese Medicine

### Analysis of Authors

**Figure 5:**
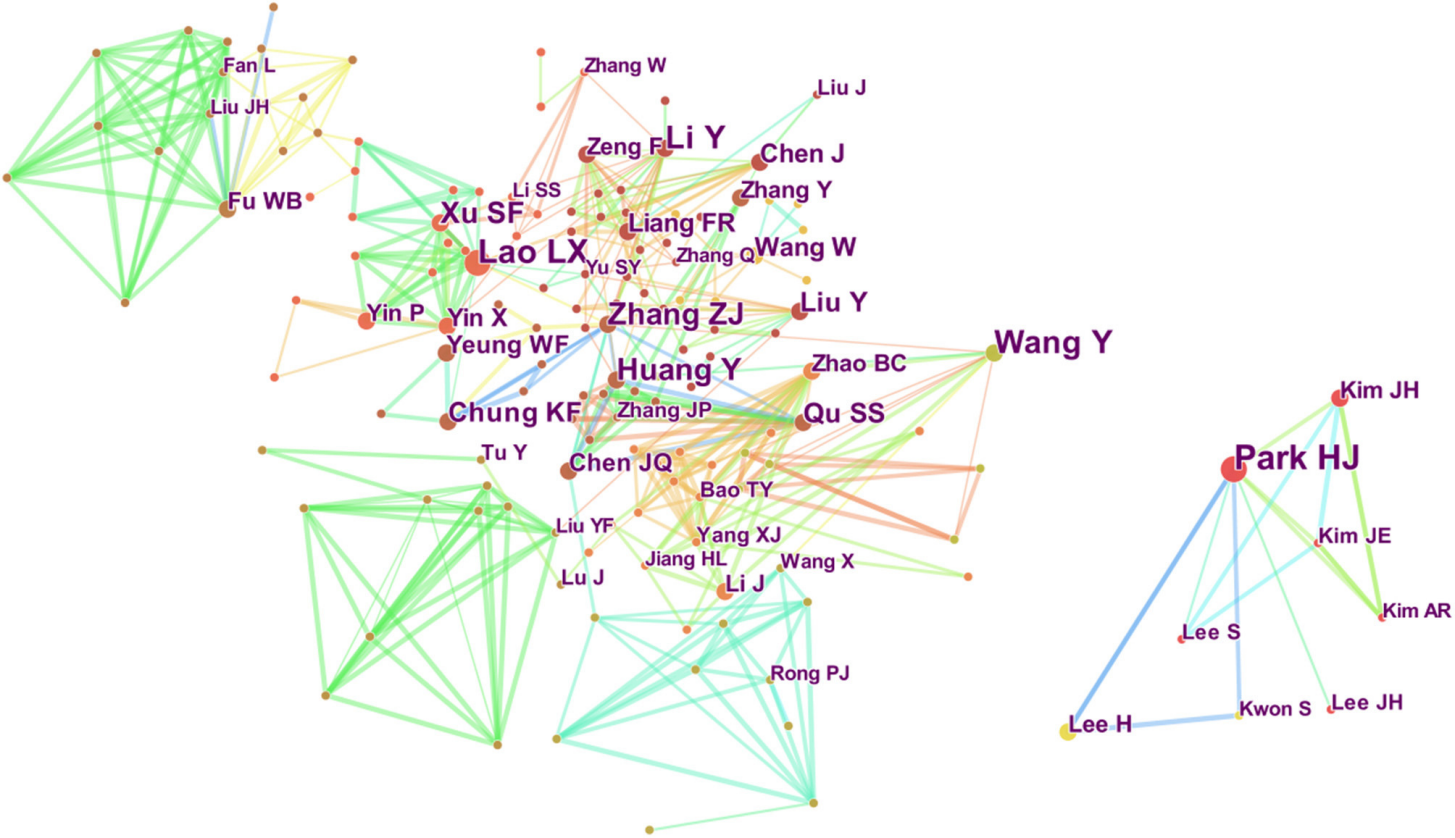
Map of co-authors related to acupuncture for depression from 2011 to 2020.

The authors of the 1,032 publications were analyzed, and 293 nodes and 741 links were generated in the author map (Figure 5). The author map showed the most prolific authors and co-authors and the links among them. It also provided information about influential research groups and potential collaborators and could help researchers establish collaborations. The top five authors in terms of the number of publications were Hi-Joon Park, Zhangjin Zhang, Lixing Lao, Yong Huang, and Shifen Xu. The top five collaborative authors in terms of centrality were Zhangjin Zhang, Lixing Lao, Yan Liu, Yue Zhang, and Shanshan Qu (Table 5). Among them, Park Hi-Joon published the most articles, from the Department of Meridian and Acupoint, College of Korean Medicine, Kyung Hee University, Seoul, South Korea. Zhangjin Zhang, who is from the University of Hong Kong, had the highest centrality among the collaborative authors. The network map (Figure 5) revealed close cooperation among the top five authors in terms of centrality, which indicated the close cooperation among these professional authors. Future collaborations among these authors would yield more studies on acupuncture for depression.

**Table 5:** Top five prolific authors and centrality of collaborative authors related to acupuncture therapy on depression.

**Rank**	**Publications**	**Author (Affiliation)**	**Rank**	**Centrality**	**Collaborative author (Affiliation)**
1	25	Hi-Joon Park (Kyung Hee University)	1	0.13	Zhangjin Zhang (University of Hong Kong)
2	22	Zhangjin Zhang (University of Hong Kong)	2	0.11	Lixing Lao (University of Hong Kong)
3	21	Lixing Lao (University of Hong Kong)	3	0.11	Yan Liu (Capital Medical University)
4	16	Yong Huang (Southern Medical University)	4	0.1	Yue Zhang (China Academy of Chinese Medical Sciences)
5	16	Shifen Xu (Shanghai University of Traditional Chinese Medicine)	5	0.09	Shanshan Qu (Southern Medical University)

### Analysis of Cited References

**Figure 6:**
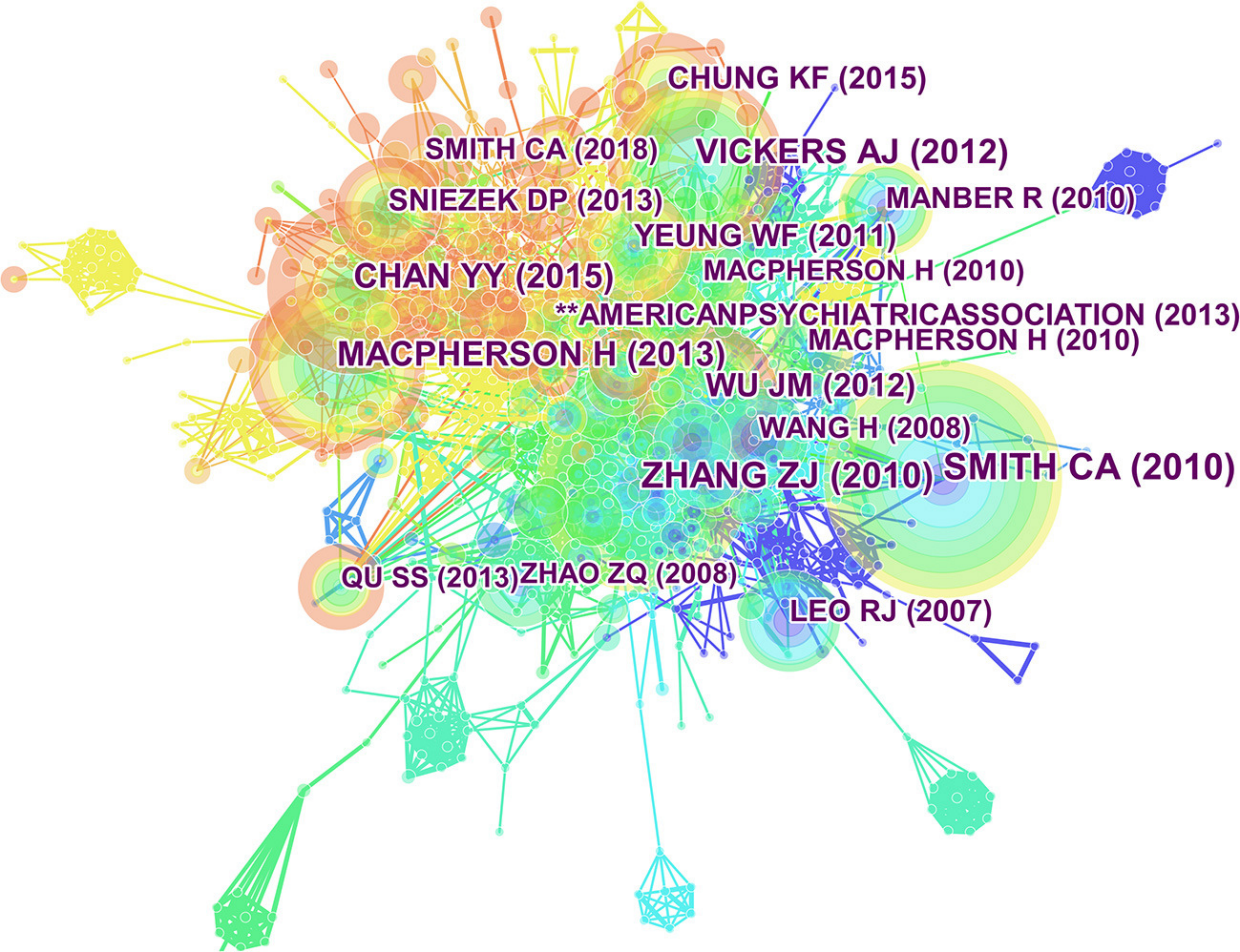
Map of cited references related to acupuncture for depression from 2011 to 2020.

The citation map comprised 778 nodes and 2,493 links (Figure 6), and the selection criteria were top 50 per slice (Timespan: 2011–2020, slice length = 1 year). Tables 6, 7 lists the top 10 publications in terms of co-citation frequency and centrality. By analyzing co-citation frequency and centrality, this study provided fundamental data on the research on acupuncture for depression. Notably, the top three highest-centrality papers were all reviews of works of literature.

**Table 6:** Top 10 frequencies of cited references related to acupuncture on depression.

**Rank**	**Frequency**	**References**	**Source**	**Author and Publication Year**
1	43	Acupuncture for depression (Smith et al., 2010)	Cochrane Database of Systematic Reviews	Smith et al., 2010
2	43	The effectiveness and safety of acupuncture therapy in depressive disorders: Systematic review and meta-analysis (Zhang et al., 2010)	Journal of Affective Disorders	Zhang et al., 2010
3	29	Acupuncture and counselling for depression in primary care: a randomised controlled trial (MacPherson et al., 2013)	Journal of Affective Disorders	MacPherson et al., 2013
4	25	Acupuncture for chronic pain: individual patient data meta-analysis (Vickers et al., 2012)	Archives of Internal Medicine	Vickers et al., 2012
5	24	The benefit of combined acupuncture and antidepressant medication for depression: a systematic review and meta-analysis (Chan et al., 2015)	Journal of Affective Disorders	Chan et al., 2015
6	23	Acupuncture for depression: a review of clinical applications (Wu et al., 2012)	Canadian Journal of Psychiatry	Wu et al., 2012
7	19	Electroacupuncture for residual insomnia associated with major depressive disorder: a randomized controlled trial (Yeung et al., 2011)	Sleep	Yeung et al., 2011
8	18	Acupuncture for residual insomnia associated with major depressive disorder: a placebo- and sham-controlled, subject- and assessor-blind, randomized trial (Chung et al., 2015)	Journal of Clinical Psychiatry	Chung et al., 2015
9	18	Acupuncture for treating anxiety and depression in women: a clinical systematic review (Sniezek and Siddiqui, 2013)	Medical Acupuncture	Sniezek and Siddiqui, 2013
10	17	A systematic review of randomized controlled trials of acupuncture in the treatment of depression (Leo and Ligot, 2007)	Journal of Affective Disorders	Leo and Ligot, 2007

**Table 7:** Top 10 centrality of cited references related to acupuncture on depression.

**Rank**	**Centrality**	**References**	**Source**	**Author and Publication Year**
1	0.25	Acupuncture for depression (Smith et al., 2010)	Cochrane Database of Systematic Reviews	Smith et al., 2010
2	0.24	Acupuncture for depression: a review of clinical applications (Wu et al., 2012)	Canadian Journal of Psychiatry-Revue	Wu et al., 2012
3	0.16	The effectiveness and safety of acupuncture therapy in depressive disorders: systematic review and meta-analysis (Zhang et al., 2010)	Journal of Affective Disorders	Zhang et al., 2010
4	0.15	Revised STandards for Reporting Interventions in Clinical Trials of Acupuncture (STRICTA): extending the CONSORT Statement (MacPherson et al., 2010)	PLoS Medicine	MacPherson et al., 2010
5	0.14	Acupuncture and counselling for depression in primary care: a randomised controlled trial (MacPherson et al., 2013)	PLoS Medicine	MacPherson et al., 2013
6	0.14	Acupuncture for depression during pregnancy a randomized controlled trial (Manber et al., 2010)	Obstetrics and Gynecology	Manber et al., 2010
7	0.12	The benefit of combined acupuncture and antidepressant medication for depression: a systematic review and meta-analysis (Chan et al., 2015)	Journal of Affective Disorders	Chan et al., 2015
8	0.11	Acupuncture for cancer-related fatigue in patients with breast cancer: a pragmatic randomized controlled trial (Molassiotis et al., 2012)	Journal of Clinical Oncology	Molassiotis et al., 2012
9	0.11	Acupuncture for the treatment of insomnia (Zhao, 2013)	International Review of Neurobiology	Zhao, 2013
10	0.09	Combination of acupuncture and fluoxetine for depression: a randomized, double-blind, sham-controlled trial (Zhang et al., 2009)	Journal of Alternative and Complementary Medicine	Zhang et al., 2009

### Analysis of Keywords

**Figure 7:**
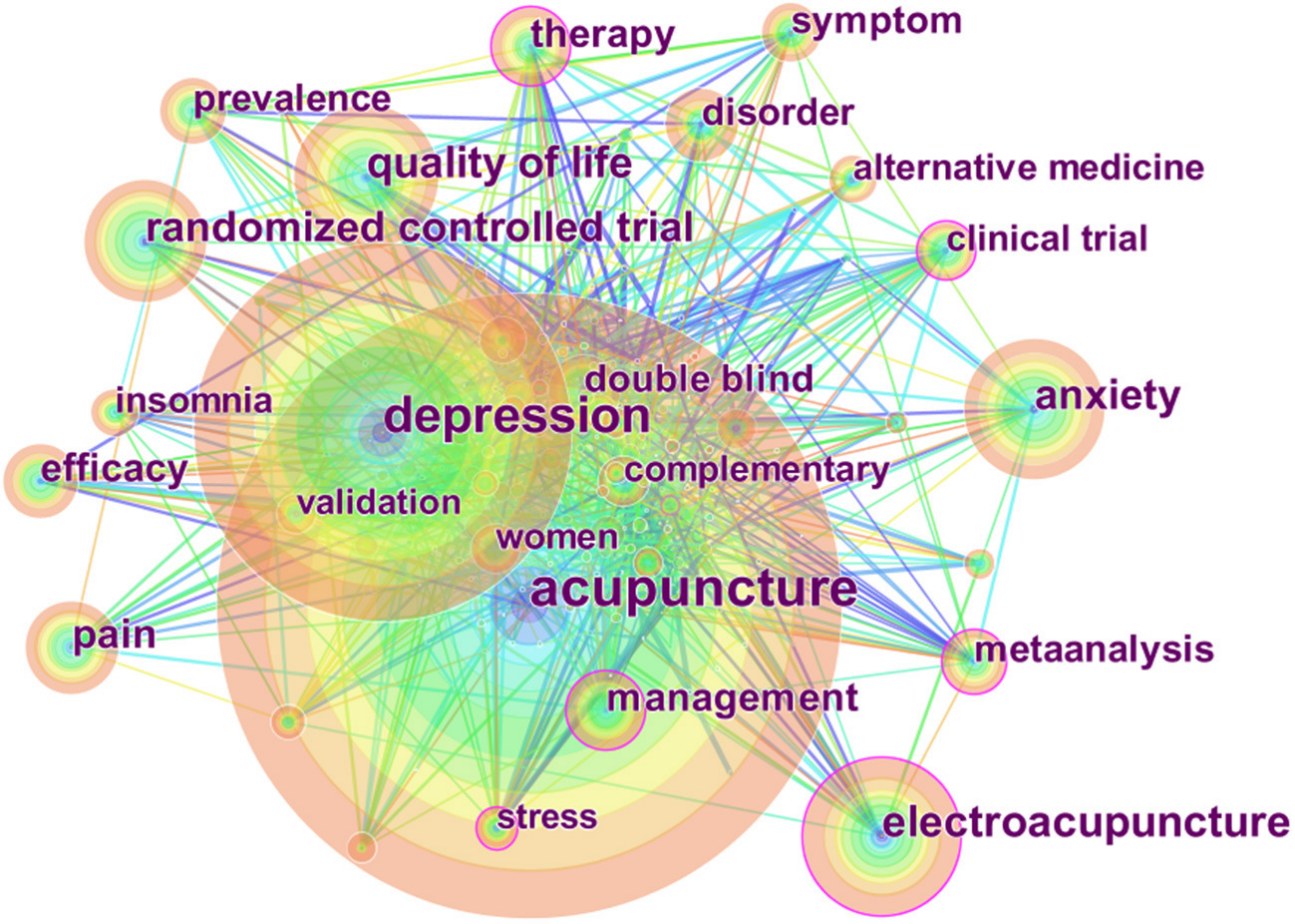
Map of keyword occurrences related to acupuncture for depression from 2011 to 2020.

**Figure 8:**
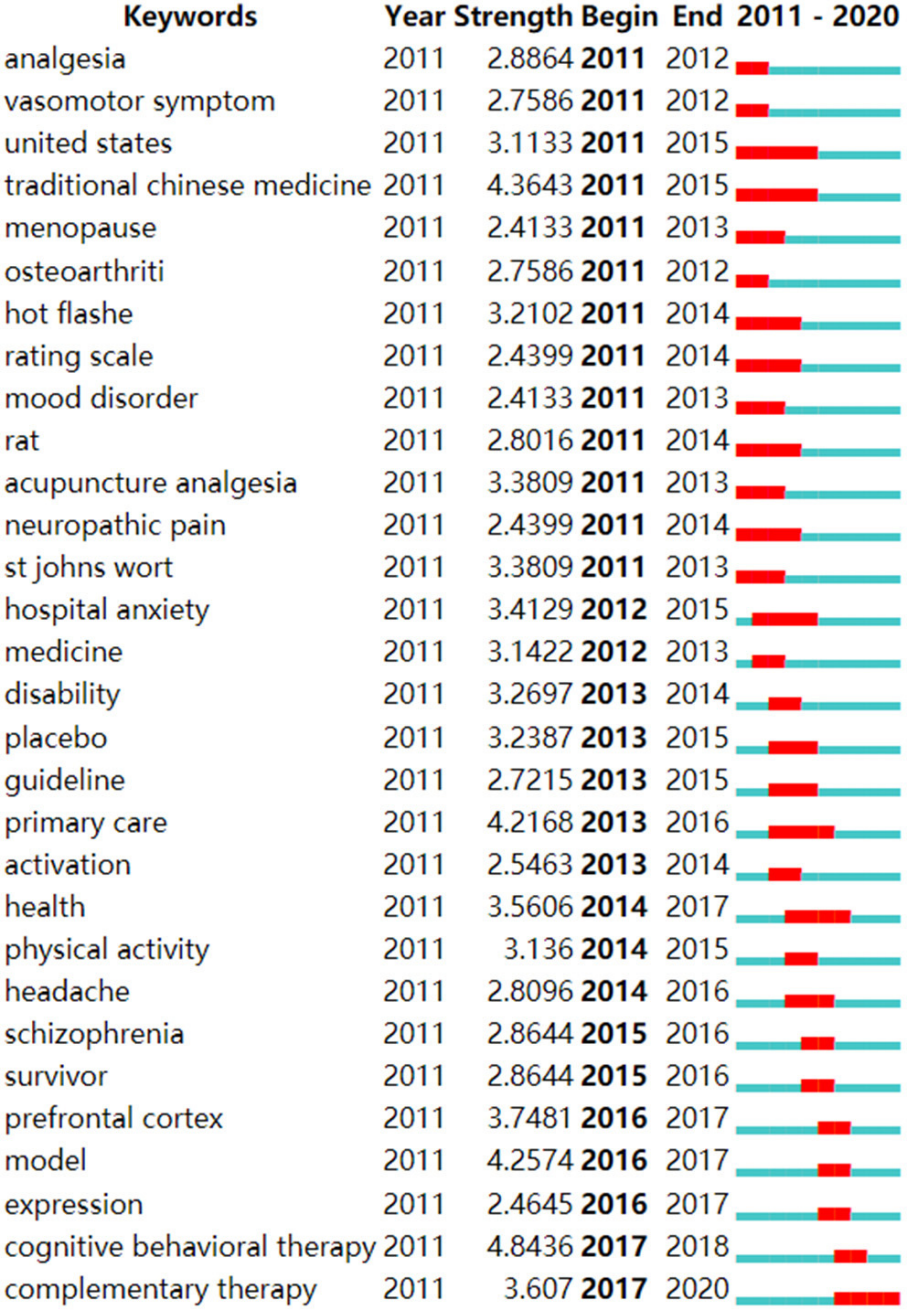
Top 30 keywords with the strongest citation bursts. The red bar indicates that the keyword was frequently referenced, and the green bar indicates that the keyword was rarely referenced.

The map of keyword co-occurrence consisted of 215 nodes and 849 links (Figure 7). The top five most frequently used keywords were “acupuncture,” “depression,” “electro-acupuncture,” “quality of life,” and “anxiety.” The top five keywords in terms of centrality were “clinical trial,” “electroacupuncture,” “stress,” “meta-analysis,” and “therapy.” We graphed the top 30 keywords in terms of citation bursts from 2011 to 2020 (Figure 8). “Burst words” are keywords frequently used within a given period of time. As shown in Figure 8, the keywords associated with citation bursts first appeared in 2011. The top five burst keywords were “cognitive behavioral therapy,” “traditional Chinese medicine,” “model,” “primary care,” and “prefrontal cortex.”

## Discussion

We performed a bibliometric analysis in the area of acupuncture for treating depression with CiteSpace by searching the Web of Science Core Collection from 2011 to 2020. We then summarized the general information and research trends in this area.

Acupuncture, as a form of traditional Chinese medicine, is one of the oldest complementary therapies (Comachio et al., 2015). Acupuncture has been used for more than 3,000 years in China and is an important component of traditional Chinese medicine (Zhuang et al., 2013). In the past 3 years, the number of publications on acupuncture treatment for depression has increased rapidly. Trend analysis indicated that acupuncture has great potential as a complementary therapy.

The most prolific countries in terms of research on acupuncture for depression are China and the United States. Acupuncture for depression is now widely accepted and actively researched in China in terms of the number of publications. Meanwhile, the United States had the highest centrality ranking for the period 2011 to 2020 and was the second-ranked country in terms of the number of publications. In the past few decades, acupuncture has increased in popularity in the United States, and its positive effects on pain, pregnancy, depression, etc., have been confirmed (Bishop et al., 2019). Four of the top five institutions in terms of the number of publications were in China, as were the top two institutions in terms of centrality. This indicates that China maintains a high degree of cooperation with other countries and institutions engaged in the research on acupuncture treatment for depression. Collaborations help researchers share information and ideas, which is very important for further development in the field of acupuncture treatment for depression. Therefore, closer cooperation networks should be established among more countries, institutions, and authors.

Zhangjin Zhang, from the School of Chinese Medicine, LKS Faculty of Medicine, the University of Hong Kong, Hong Kong, China, had the highest centrality (Zhang et al., 2020a). Professor Zhang published many research papers on randomized controlled trials of the acupuncture treatment for depression. A randomized controlled trial by this author showed that, compared with minimum acupuncture stimulation, dense cranial electroacupuncture stimulation plus body acupuncture can significantly alleviate post-stroke depression, functional disability, and the cognitive deterioration of stroke patients, but the subjects of this study included ischemic and hemorrhagic stroke, so their pathological characteristics and symptom severity may be different (Zhang et al., 2020b). In a single-blind randomized controlled study, Zhang-Jin Zhang showed that dense cranial electroacupuncture stimulation plus SSRIs significantly alleviated major depression after 3 weeks based on the 17-item Hamilton Depression Rating Scale compared with SSRIs alone in the early phase (Zhang et al., 2012).

The author with the most publications was Park Hi-Joon, from the Department of Meridian and Acupoint, College of Korean Medicine, Kyung Hee University, Seoul, South Korea (Lee et al., 2014). In recent years, professor Park Hi-Joon has published papers about acupuncture that are not directly related to depression. For instance, one study was on the diagnostic principles and acupoint selection for patients with functional dyspepsia. Patients were classified into “spleen-stomach weakness,” “liver qi depression,” and “food accumulation or phlegm-fluid retention” groups. These subclasses have both common and divergent acupoints (Kim et al., 2020). A randomized controlled trial, a coping strategy, a questionnaire, Beck's depression inventory, and the State-Trait Anxiety Inventory were used to evaluate the therapeutic effects of acupuncture on chronic sciatica (Kim et al., 2019). In another randomized controlled trial, the feasibility and efficacy of acupuncture for atopic dermatitis (including pruritus) were evaluated using instruments including the Center for Epidemiologic Studies Depression Scale (Kang et al., 2018). These studies were not to clarify the efficacy and mechanism of acupuncture in the treatment of depression but to use the depression assessment scale.

In addition, the centrality of Lao Lixing ranked second. He is from the School of Chinese Medicine, the University of Hong Kong (Yin et al., 2019). A systematic review by this author showed that acupuncture combined with Western medicine is more effective for improving sleep quality than Western medicine alone. Acupuncture for depression-related insomnia may be an alternative to drug therapy (Dong et al., 2017). A randomized controlled trial by Lao Lixing showed that electroacupuncture combined with antidepressants was more effective than antidepressants alone for improving depressive symptoms, possibly due to its effects on tryptophan metabolism, glutamate metabolism, and fatty acid biosynthesis (Li W. et al., 2020).

The top three highest co-citation centrality papers were all reviews. The first review found insufficient evidence to support the view that acupuncture is ineffective for depression, mainly due to the high risk of bias in most trials meeting the inclusion criteria (Smith et al., 2010). In the second review, acupuncture is a potentially effective monotherapy for the treatment of depression. It is a safe and well-tolerated enhancement therapy for partial responders and non-responders against depression (Wu et al., 2012). In the third review, the acupuncture group had superior outcomes to antidepressant and waiting list control groups in terms of post-stroke depression and symptom severity. When compared with antidepressants alone, the superior effect of acupuncture combined with antidepressants only appears in improving depressive symptoms rather than clinical responses in terms of major depressive disorder. This unexpected result may be due to the limited number of trials and small samples available for analysis, resulting in the insufficient ability to detect statistical significance (Zhang et al., 2010).

The five most frequently used keywords were “acupuncture,” “depression,” “electro-acupuncture,” “quality of life,” and “anxiety.” The five keywords in terms of centrality were “clinical trial,” “electroacupuncture,” “stress,” “meta-analysis,” and “therapy.” Complementary and alternative medicine has become a promising option for the treatment of depression, such as the use of medicinal herbs and acupuncture, etc. (Haller et al., 2019). In a pragmatic randomized controlled trial, pregnant women were treated with acupuncture at 24–31 weeks of gestation. Clinical evaluations were performed throughout the intervention and at a 6-week follow-up. Prenatal acupuncture reduced depression, stress, and pain and improved the quality of life without adverse reactions (Ormsby et al., 2020). A systematic review and meta-analysis demonstrated the efficacy of acupuncture in the treatment of postpartum depression (PPD). The Hamilton Depression Scale of the acupuncture group was significantly better than those of the control group, and efficacy was significantly higher for the acupuncture than the control group. However, in a subgroup analysis, only the efficacy rate of acupuncture remained superior (Tong et al., 2019). Another meta-analysis evaluates the effect of acupuncture on post-stroke depression. There was a significant difference between the acupuncture alone and standard medicine groups, but not between the acupuncture combined with standard medicine and standard medicine groups (Zhang et al., 2019). An overview of meta-analyses has shown that acupuncture treatment is more effective and safer than non-treatment, sham acupuncture, and antidepressants. A subgroup analysis revealed no difference between electroacupuncture and invasive control groups, electroacupuncture and non-invasive control groups, hand acupuncture and tetracycline antidepressant groups, or electroacupuncture and tetracycline antidepressant groups (Li M. et al., 2020). However, the meta-analyses had some limitations, including a small number of cases and high heterogeneity.

Bibliometric analysis can show the global research trends and identify gaps in the extant literature (Mulet-Forteza et al., 2020), but there are still some limitations to this study. First, we only analyzed publications in the Web of Science database, which may lead to a language and publishing bias. In the future, China National Knowledge Infrastructure (CNKI) data should also be analyzed. Second, using CiteSpace, more types of depression, acupuncture methods, and diseases need to be analyzed in the future.

## Conclusion

In summary, this study provided useful data for potential collaborations among researchers and institutions and identified hot topics and trends in the research on acupuncture treatment for depression. Acupuncture appears effective in the treatment of depression, but more evidence is still required. Therefore, further research is needed, following standardized guidelines and with a low risk of bias, including high-quality randomized controlled trials.

## Author Contributions

GC and HX designed the ideas of the paper. QT and BL collected the data from Web of Science. HX and JL analyzed the data. HX drafted the manuscript. All authors read and approved the final manuscript.

## Funding

This study was supported by the Natural Science Foundation of China (No. 81774410).

## Conflict of Interest

The authors declare that the research was conducted in the absence of any commercial or financial relationships that could be construed as a potential conflict of interest.

## Publisher's Note

All claims expressed in this article are solely those of the authors and do not necessarily represent those of their affiliated organizations, or those of the publisher, the editors and the reviewers. Any product that may be evaluated in this article, or claim that may be made by its manufacturer, is not guaranteed or endorsed by the publisher.
